# Formation of Surface and Quantum-Well States in Ultra Thin Pt Films on the Au(111) Surface

**DOI:** 10.3390/ma10121409

**Published:** 2017-12-09

**Authors:** Igor V. Silkin, Yury M. Koroteev, Pedro M. Echenique, Evgueni V. Chulkov

**Affiliations:** 1 Department of Physics, Tomsk State University, 634050 Tomsk, Russia; yury_koroteev@list.ru; 2 Institute of Strength Physics and Materials Science, Siberian Branch, Russian Academy of Sciences, 634050 Tomsk, Russia; 3 Department of Physics, Saint Petersburg State University, 198504 Saint Petersburg, Russia; 4 Donostia International Physics Center (DIPC), 20018 San Sebastian/Donostia, Basque Country, Spain; wapetlap@ehu.eus (P.M.E.); evguenivladimirovich.tchoulkov@ehu.eus (E.V.C.); 5 Department of Materials Physics, Materials Physics Center CFM-MPC and Mixed Center CSIC-UPV, 20080 San Sebastian/Donostia, Basque Country, Spain

**Keywords:** gold, platinum, surface states, quantum-well states, spin splitting, heterostructure

## Abstract

The electronic structure of the Pt/Au(111) heterostructures with a number of Pt monolayers *n* ranging from one to three is studied in the density-functional-theory framework. The calculations demonstrate that the deposition of the Pt atomic thin films on gold substrate results in strong modifications of the electronic structure at the surface. In particular, the Au(111) *s*-*p*-type Shockley surface state becomes completely unoccupied at deposition of any number of Pt monolayers. The Pt adlayer generates numerous quantum-well states in various energy gaps of Au(111) with strong spatial confinement at the surface. As a result, strong enhancement in the local density of state at the surface Pt atomic layer in comparison with clean Pt surface is obtained. The excess in the density of states has maximal magnitude in the case of one monolayer Pt adlayer and gradually reduces with increasing number of Pt atomic layers. The spin–orbit coupling produces strong modification of the energy dispersion of the electronic states generated by the Pt adlayer and gives rise to certain quantum states with a characteristic Dirac-cone shape.

## 1. Introduction

The heterostructures consisting of the Au, Ag, Cu noble metal substrate and the late d-metal adsorbate—Ir, Ni, Pt, Pd and other heavy elements with the degree of coverage from one atom to several monolayers (MLs)—have been actively studied for the last two decades [1,2,3,4,5,6,7,8,9,10,11,12,13,14]. The interest to these systems can be explained by the possibility of exploiting them in chemical industry, namely, in heterogeneous catalysis. It is known that the properties of two-dimensional systems can drastically differ from the properties of their bulk counterparts due to structural and electronic effects [15,16]. Frequently, the degree of coverage plays a crucial role in these effects. A striking example of such situation is the Pt/Au(111) heterostructure with the Pt adsorbate thickness varying from one to several atomic layers. In general, favorable catalytic activity, resulting in strong enhancement in rates of certain oxidation reactions, of thin Pt films deposited on Au has been found [17,18,19,20,21,22,23,24,25,26,27,28].

On the other hand, heterostructures containing as a substrate a heavy metal and several atoms/monolayers of metallic adsorbate demonstrate unique properties caused by spin–orbit interaction that may be attractive for spintronics, a research field experiencing explosive development nowadays. A well-known example is the Bychkov–Rashba splitting effect consisting of lifting of a spin degeneration in two-dimensional systems due to spin–orbit interaction [29]. Large Bychkov–Rashba splitting is characteristic for surface states of both noble and late *d*-metals such as Au and Pt, and other heavy metals [30,31,32,33,34,35,36,37,38,39]. Moreover, the heterostructures based on light noble metals (Cu and Ag) and heavy metal adsorbate such as Bi and Pb have a giant spin–orbit splitting of surface states noticeably larger than in pristine materials [40,41,42,43]. The main reason for this giant splitting is the occurrence of a local potential gradient at the surface of such heterostructures that does not exist inside the bulk materials with inversion symmetry.

In atomically thin films, the spatial confinement in the direction perpendicular to the film surface leads to the quantization of the electronic states. The resulting quantum-well states (QWSs) [15,44,45,46,47,48] were intensively studied during last decades. It was demonstrated that many properties of thin films may depend of their thickness due to modulation of the density of states at the Fermi level. This effect was mainly investigated in films consisting of simple metals [49,50,51,52,53,54,55,56,57,58,59,60,61,62,63,64,65,66,67].

Frequently, the quantization of electronic states in thin films in the direction perpendicular to surface can be understood rather well in the framework of a semiclassical description based on a Fabry–Perot-type approach [15]. Even though the potential corrugation at the interatomic scale caused by the atoms can have some effect [68,69,70], this basic picture for description of quantization of the *s*-*p* electronic states still can be valid. However, many important materials have an electronic structure being far different from a quasi-free-electron-like picture. For instance, a proper description of localized *d*-like electronic states is possible only employing the first-principles calculation methods. In particular, nowadays, the density functional method allows studying in detail the atomic and electronic structure of almost any moderately correlated system. This is particularly important for materials having the *d* electrons at the Fermi level since, in catalytic materials, the number of charge carriers at the Fermi level plays an important role, the large density of them ensuring a higher reaction rate and strength of catalytic reactions [71].

In this work, we investigate the formation of electronic states in thin Pt films deposited on the Au(111) substrate. From comparison of the electronic structure of the Pt(111) and Au(111) surfaces, one can observe a strong mismatch between positions of the band gaps in the projected bulk electronic structures of these materials. As a result, the electrons related to platinum atoms are reflected from the gold substrate by its energy gaps. Together with scattering produced by the potential barrier from the vacuum side, this introduces the necessary conditions for realization of the QWSs. Since the electronic states in Pt in the Fermi level vicinity are mainly of the *d* character, they present strong localization at the surface. In turn, this produces strong modification in the density of states in the surface region. Studying Pt-derived QWSs at different regions of the surface Brillouin zone (SBZ), we find distinct formation character. We also investigate in the Pt/Au(111) heterostructures the effect of spin–orbit interaction on the electronic states localized at the surface since despite many experimental and theoretical studies devoted to these systems with adsorbate thickness of several MLs, the effect of the spin–orbit interaction on the electronic structure of these systems was not addressed.

The rest of the paper is organized as follows: in Section 2, we describe some details of the ab initio calculation of the surface electronic structure in the slab configuration. The calculated results and their discussion are reported in Section 3. The main conclusions of this work are presented in Section 4. Unless otherwise stated explicitly, atomic units ($\hbar =e^{2}=m_{e}=1$) are used throughout the paper.

## 2. Calculation Method

The calculations were performed within the framework of the density functional theory employing a pseudopotential method implemented in the VASP (Vienna Ab-initio Simulation Package) code [72,73]. For description of the exchange-correlation effects, the local density approximation was used in the Ceperly–Alder parametrization [74].

The 5d${}^{10}$6s${}^{1}$ and 5d${}^{9}$6s${}^{1}$ configurations for the valence electrons in Au and Pt, respectively, were used. Self-consistency of the electron density was carried out on a 11 × 11 × 1 grid of k-points constructed by the Monkhorst–Pack scheme [75]. For all calculations, we used the criterion of convergence with respect to the total energy to $10^{-8}$ eV.

The optimized bulk lattice parameters are $a_{0}$ = 4.04 Å for Au and $a_{0}$ = 3.90 Å for Pt. The calculated lattice constants are in good agrement with the experimentally determined values $a_{0}$ = 4.045 Å [76] for Au and $a_{0}$ = 3.924 Å [77] for Pt. The clean Pt(111) and Au(111) surfaces of a semifinite crystal were simulated with a 23-layer film. The same number of layers was employed for the description of the gold substrate in the case of *n*ML-Pt/Au(111) systems, where the Pt adlayers consisting of *n* atomic layers were placed on both sides of the slab. The in-plane lattice parameter for the Pt adlayers was chosen to be equal to the Au bulk constant. For each system, we performed optimization of the vertical atomic positions in the Pt adsorbate layers and four outer layers of the Au substrate on each side of the film. Atomic positions of fifteen internal Au atomic layers were frozen. In order to estimate a possible effect of the lateral lattice contraction in Pt monolayers, we calculated the electronic structure of free-standing 3 ML Pt films with the Pt and Au lateral lattice parameters. The energies of the Pt QWSs in both calculations differ less than 0.15 eV. The same modification of energies of these states can also be expected in Pt/Au(111) systems.

## 3. Results and Discussion

### 3.1. Clean Pt(111) Surface

**Figure 1:**
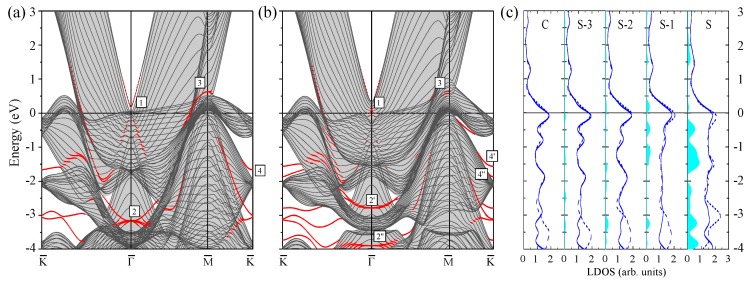
The electronic structure of the Pt(111) surface calculated for the 23 monolayer thick slab without (**a**) and with (**b**) spin–orbit interaction. The surface states and resonances are indicated by red dots. The surface states discussed in the text are labeled by symbols. In (**a**) and (**b**), the continuum of the projected bulk electronic states is indicated by the grey color. In (**c**), the layer density of states (LDOS) is shown for the top four (labeling of atomic layers starts from the surface (S) one) and the central (C) atomic layers. Solid (dashed) lines show LDOS obtained from the calculation with (without) spin-orbit coupling. Blue regions show the excess of LDOS in a given layer in comparison with the bulk values.

Since a limiting case of a large number of the atomic layers in a Pt slab is a clean Pt(111) surface, we start with presentation of its calculated electronic structure in Figure 1 for the reference. In panels (a) and (b), the Pt(111) surface electronic structures calculated without (WSOC) and with (SOC) inclusion of the spin–orbit coupling, respectively, are shown. The figure demonstrates that the bulk electronic states of mainly *d* character dominate the electronic structure of this material at the Fermi level. Such states intersect the Fermi level in all symmetry directions of the SBZ. This is explained by the presence in Pt of a partly filled valence *d* band containing only nine electrons. On the other hand, the electronic states at energies above ≈0.5 eV are of mainly *s*-*p* character.

**Figure 2:**
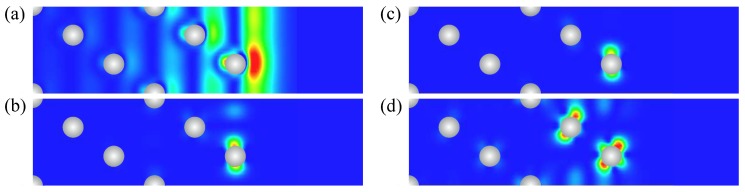
Charge density distribution of (a) $\Gamma_{1}$, (b) $\Gamma_{2}$, (c) $M_{3}$, (d) $K_{4}$ surface states on clean Pt(111) surface obtained from the WSOC calculation. The Pt atomic positions are shown by grey dots.

In the WSOC projected bulk electronic continuum shown in grey in Figure 1a, there are several energy gaps in which one can observe surface states with wave functions localized at the surface and decaying in both the vacuum and the crystal inside directions. Thus, above the Fermi level around the $\bar{\Gamma }$ point, a totally unoccupied *s*-*p*-type surface state $\Gamma_{1}$ characterized by a free-electron-like up-ward dispersion resides in a wide energy gap. It is located very close to the upper edge of the projected bulk states continuum and its energy position at $\bar{\Gamma }$ is 0.17 eV above the Fermi level. The charge density distribution of this surface state presented in Figure 2 confirms its spatial confinement to the surface region although its penetration into the crystal is significant and is notable even in the fifth atomic layer. Such empty *s*-*p* surface state was observed on the (111) surfaces of Pt [78,79,80] and Pd [81,82,83]. On the contrary, we do not find any surface state in the energy gap at the $\bar{K}$ point at energies above −0.1 eV.

It can be deduced from Figure 1a that, in the WSOC case, in the occupied part of the spectrum there are surface states of the *d* type along all symmetry directions. Thus, at $\bar{\Gamma }$ in a symmetry energy gap for the *d* states extending from −3.47 to −1.78 eV, we find a double degenerate surface state $\Gamma_{2}$ with energy of −3.1 eV. As seen in its charge density distribution of Figure 2, it has a $d_{xy}$, $d_{x^{2}-y^{2}}$ character and its wave function is strongly localized at the surface atomic layer. When the wave vector moves away from the $\bar{\Gamma }$ point, this state splits into two energy bands [84] with the positive and negative dispersions, respectively. The upper energy band $\Gamma_{2}$ seen in Figure 1a may be clearly resolved up to energy of −2.6 eV along the $\bar{\Gamma }\;\bar{M}$ direction, whereas along the $\bar{\Gamma }\;\bar{K}$ direction it becomes a resonance at energies above around −2.3 eV. This resonance state can be traced in this symmetry direction up to energies exceeding 1 eV above the Fermi level.

In the vicinity of the $\bar{M}$ point, we find the surface state $M_{3}$ above the Fermi level. Despite lack of the energy gaps in the WSOC projected bulk electronic structure in this energy interval, this surface state presents strong localization at the surface atoms as seen in Figure 2. At the $\bar{K}$ point, we find surface states in the occupied part of the WSOC surface electronic structure. In the energy interval of interest, we observe here an occupied surface state denoted as $K_{4}$ at −1.66 eV in one of the energy gaps. This surface state localizes in the two upper surface layers (see Figure 2). The orbital composition of these surface states is a mixture of $d_{xy}$, $d_{xz}$, $d_{yz}$, and $d_{x^{2}-y^{2}}$ orbitals. Upon moving from the $\bar{K}$ point, the $K_{4}$ surface state disperses upward and quickly disappears reaching the energy gap edges.

**Figure 3:**
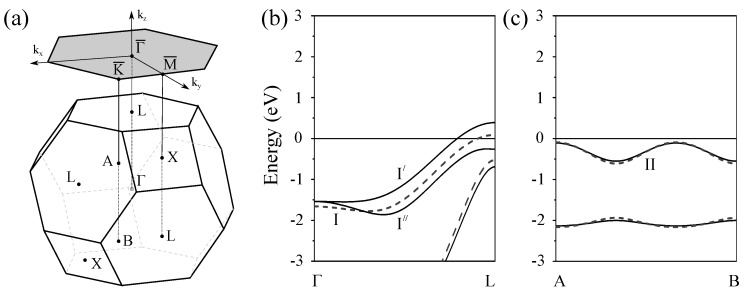
(**a**) bulk Brillouin zone and its projection onto the (111) surface. Bulk electronic structure of Pt along the (**b**) ΓL and (**c**) AB directions of the bulk Brillouin zone [38], determining the Au(111) projected bulk states continuum atthe $\bar{\Gamma }$ and $\bar{K}$ points of the SBZ, respectively. The solid (dashed) lines present energy bands calculated with (without) inclusion of the spin–orbit coupling. One can see how upon inclusion the spin–orbit coupling a four-fold degenerate band I splits in two spin-degenerate states I${}^{\prime }$ and I${}^{\prime \prime }$ along ΓL. Instead, the other bands, including the band II of interest here, are barely affected by the spin–orbit interaction.

Switching on the spin–orbit interaction substantially changes the calculated surface electronic structure of Pt(111) as follows from comparison of Figure 1b with Figure 1a. In particular, significant modifications are observed in the dispersion of the energy bands representing the projected bulk states. Thus, the lower edge of the energy gap above the Fermi level at the $\bar{\Gamma }$ point shifts upward and locates at 0.35 eV in the SOC case. It is accompanied by a significant downward shift of the other bulk band forming this edge in the WSOC case to around −0.25 eV, which results in a dip in the $\bar{\Gamma }$ point vicinity. This behavior is explained by the strong spin–orbit splitting [85,86,87] of the upper energy band along the ΓL symmetry direction of the bulk Brillouin zone presented in Figure 3a. In Figure 3b, one can see how the upper WSOC double-degenerate band I with energy of 0.09 eV above the Fermi level at L splits in the SOC case into two energy bands I’, I” with energies of 0.39 and −0.26 eV at L. At the same time, its dispersion in the Γ point vicinity is hardly affected by the spin–orbit coupling as well as in the case of the lower energy band of mainly *s*-*p* character.

Moreover, upon inclusion of the spin–orbit coupling, the *s*-*p*-type unoccupied surface state $\Gamma_{1}$ splits into two spin-resolved bands. In the SOC calculation, these energy bands shift upward and locate at around 0.35 eV at the $\bar{\Gamma }$ point. These modifications are accompanied by loosing the surface-state-like character in the close vicinity of the $\bar{\Gamma }$ point. Instead, the energy bands having energy of 0.11 eV at $\bar{\Gamma }$ acquire a clear surface state behavior with its charge density distribution similar to that reported in Figure 2. It signals that the surface state band $\Gamma_{1}$ penetrates slightly into the continuum of projected bulk states caused by a notable spin–orbit splitting of the four-fold degenerate bulk energy band determining the lower edge of the energy gap. Thus, in Figure 3b, the upper edge of the top spin–orbit-split energy band at the bulk *L* point determining the energy position of the lower edge of the gap is at 0.39 eV. In the case of Pt(111), the observed strong hybridization of the $\Gamma_{1}$ surface state with bulk states impedes determination of Bychkov–Rashba splitting parameter $\alpha_{R}$, which agrees with References [38,80].

In Figure 1b, one can see how the surface states of the *d* character lying in the occupied part of the spectrum experience substantial splitting and modification due to the spin–orbit interaction. The effect of spin–orbit interaction on the surface state $\Gamma_{2}$ is rather strong. Thus, at $\bar{\Gamma }$, it splits into two bands $\Gamma_{2^{\prime }}$ and $\Gamma_{2^{\prime \prime }}$ with energies of −2.8 and −3.8 eV, respectively. Due to such a splitting, the energy dispersion of the $\Gamma_{2^{\prime }}$ band is significantly more shallow in comparison with that of the WSOC $\Gamma_{2}$ one around the $\bar{\Gamma }$ point. At the same time, its dispersion along both symmetry directions at finite wave vectors propagates in fairly the same energy window.

In the SOC calculation, we could not resolve any clear surface state at the $\bar{M}$ point. The effect of the spin–orbit coupling on the surface state at the $\bar{K}$ point is significant despite its little effect of the bulk electronic structure along the AB direction seen in Figure 3c. As a result, the surface state $K_{4}$ (with energy of −1.66 eV in the WSOC case) splits into two surface state bands $K_{4^{\prime }}$ and $K_{4^{\prime \prime }}$ with energies of −1.56 and −1.86 eV, respectively. Since the upper energy spin-split state is pushed up in the energy gap it maintains its surface-state-like character over extended momentum range in comparison with the WSOC case. While the lower energy spin-split surface state $K_{4^{\prime \prime }}$ is moved down closer to the energy gap edge, it quickly disappears upon moving away from the $\bar{K}$ point along the $\bar{K}\;\bar{M}$ symmetry direction. On the contrary, along the $\bar{K}\;\bar{\Gamma }$ direction, it propagates over a larger distance due to the strongly anisotropic shape of the energy gap.

In general, our calculated SOC Pt(111) surface electronic structure is very close to that reported in reference [38], where detailed analysis of the all surface states on this surface can be found. A careful comparison of the calculated surface electronic structure of Pt(111) with the data of the photoemission [88,89] and scanning tunneling microscopy [80] measurements is given in the same publication as well.

The surface electronic states of predominantly *d*-type give rise to the strong peaks in the layer density of states (LDOS) of Figure 1c. In particular, the strong excess of the charge in the surface layer in comparison with the bulk LDOS is observed in the −1.79 to −0.19 eV interval due to the presence of the $K_{3}$ surface state. The effect of the spin–orbit coupling of LDOS is significant as well. In particular, its inclusion reduces the LDOS in the surface layer just below the Fermi level and at −3 eV. The former we relate to the disappearance of the WSOC band with strong surface localization around the $\bar{M}$ at the Fermi level. The latter is explained by spin–orbit splitting of the surface state band $\Gamma_{2}$ and states at the $\bar{K}$ point.

### 3.2. Clean Au(111) Surface

**Figure 4:**
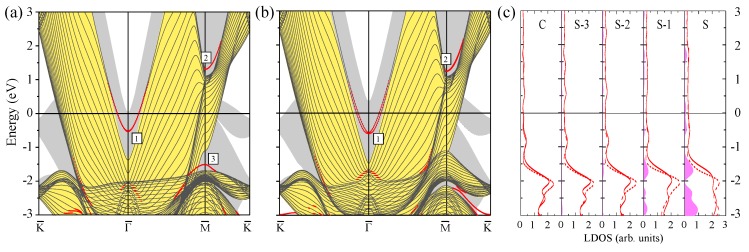
The electronic structure of the Au(111) surface calculated for the 23 monolayer thick slab without (**a**) and with (**b**) spin–orbit interaction. The surface states and resonances are indicated by red dots. The surface states discussed in the text are labeled by symbols. In (**a**) and (**b**), the continuum of the projected bulk electronic states for the Au(111) surface is shown by the yellow color while the portion of the projected bulk electronic structure for Pt(111) located outside this continuum is presented by the grey color. In (**c**), the layer density of states is shown for the top four and the central atomic layers. Pink regions show the excess of LDOS in a given layer in comparison with the bulk values.

In Figure 4a,b, we show the WSOC and SOC electronic structures of the Au(111) surface, respectively. As mentioned above, due to the fullfilling of the 5*d* energy shell, the valence *d* band of gold is completely filled and is represented in Figure 4a by the electronic states at energies below of about −2.0 eV, while the electronic states at the Fermi level are mainly of the *s*-*p* type, in contrast to what occurs in Pt. Another important difference is the energy position of the *s*-*p* energy gap at the $\bar{\Gamma }$ point. Contrary to the Pt(111) case in Au(111), it is partly located below the Fermi level.

**Figure 5:**
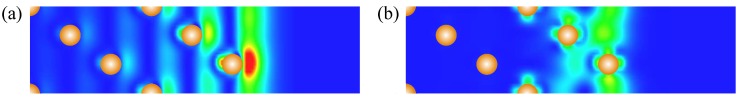
Charge density distribution of (a) $\Gamma_{1}$, (b) $M_{2}$ surface states on clean Au(111) surface obtained from the WSOC calculation. The Au atomic positions are shown by yellow dots.

In the WSOC case of Figure 4a in the calculated surface electronic structure, in addition to the projected bulk-like states continuum, there are three surface states. In an energy gap around the $\bar{\Gamma }$ point at energies above −1.4 eV, one can see a surface state $\Gamma_{1}$ with a parabolic-like upward dispersion. It is partly occupied with its bottom located at energy of −0.51 eV at $\bar{\Gamma }$. It is of a *s*-*p* type and rather strongly localized at the Au(111) surface as demonstrates its charge density distribution reported in Figure 5.

In the WSOC case, the other two surface states reside in the energy gaps in the vicinity of the $\bar{M}$ point. The *s*-*p*-like state denoted as $M_{2}$ is totally unoccupied and has upward dispersion with energy of 1.3 eV at the $\bar{M}$ point. Plot with its charge density distribution at $\bar{M}$ shown in Figure 5 confirms its strong localization in the three top Au atomic layers. The other surface state $M_{3}$ is totally occupied and has downward dispersion. It is located at energy of −1.5 eV at the $\bar{M}$ point.

Figure 4b shows the electronic structure of the Au(111) surface calculated with the spin–orbit interaction included. Here, one can see several differences in comparison with the calculated WSOC electronic structure of Figure 4a. First, the *d*-like electronic states forming the bulk-like continuum are shifted closer to the Fermi level and its upper edge is located at the Γ point at energy of −1.7 instead of −2.0 eV in the WSOC case. Second, the *s*-*p*-like surface state $\Gamma_{1}$ residing in the vicinity of the $\bar{\Gamma }$ point is shifted downward and has an energy of −0.58 eV at $\bar{\Gamma }$. Moreover, upon inclusion of the spin–orbit interaction, this state experiences notable splitting of the Bychkov–Rashba type with a splitting coefficient $\alpha_{R}$ = 0.8 eV·Å. Third, at the $\bar{M}$ point in the occupied part, the energy gap (containing surface state $M_{3}$) existing in the −1.7 to −1.1 eV interval in the WSOC case is closed in the SOC case. It is accompanied by disappearance of the surface state located in such a gap. Instead, another narrow energy gap opens at about −2.1 eV in the projected bulk electronic structure. On the contrary, the surface state $M_{2}$ lying at the $\bar{M}$ point in the unoccupied part is not affected notably upon switching on the spin–orbit interaction. The only effect is a slight spin–orbit splitting of this energy band along the $\bar{M}\;\bar{K}$ direction. In general, the Au(111) surface electronic structure calculated here is in good agreement with previously published both theoretical [34,90,91] and experimental [31,92,93,94,95,96,97,98,99,100] results.

In Figure 4c, we show LDOS obtained from the SOC calculation for the top four and central atomic layers of Au(111). It is well known that the densities of states for Au and Pt strongly differ from each other, especially at the Fermi level. For bulk gold, the density of states at the Fermi level is around 0.25 states/eV, while, for bulk platinum, it reaches a value of 1.75 states/eV. In gold, the electronic states at the Fermi level are mainly of the *s*-*p* character while the *d*-like states dominate electronic structure in this energy interval in Pt. In the case of Au(111), a sharp increase in the density of states due to the *d* band occurs at energies below ≈−1.3 eV. In Pt, this threshold caused in the density of states by the *d* states occurs at energy of about 0.5 eV in the unoccupied part.

As seen in Figure 1c, in the third and fourth atomic layers, the LDOS does not deviate significantly from that for the inner bulk-like C layer. At the same time comparing Figure 1c and Figure 4c it can be noted that in both Pt and Au the LDOS of the surface and one subsurface layers is visibly different from that of the inner layers, which can be explained by the presence of the surface electronic states.

The potential barriers at the vacuum and substrate interfaces confine the electrons in the absorbed film interior. As a result, in thin films, the bulk electronic states transform into a set of states confined to it. They are characterized by a quantized states in the direction perpendicular to the slab and a bulk-like dispersion in the plane. In Figure 4a,b, we add the projected bulk continuum states of Pt(111) in order to demonstrate its strong mismatch with the projected bulk electronic structure of Au(111). The electronic states confined to the Pt adlayers in these regions get trapped in the adlayer since its penetration into the metal is prohibited by the projected band gap of the substrate. In the following, we shall concentrate on the formation of the QWSs in three regions, namely, in the *s*-*p* partly occupied energy gap at the $\bar{\Gamma }$ point, in the totally unoccupied gap at $\bar{M}$, and in the upper gap in the vicinity of the $\bar{K}$ point.

### 3.3. n ML-Pt/Au(111) Heterostuctures

#### 3.3.1. 1 ML-Pt/Au(111)

**Figure 6:**
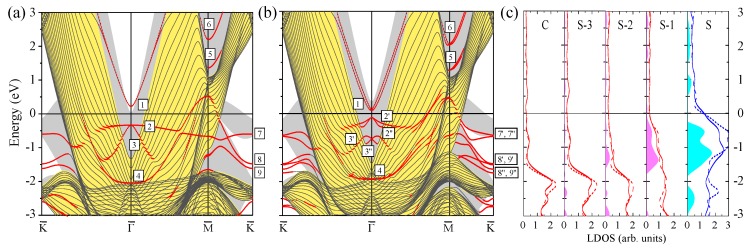
The electronic structure of the 1 ML-Pt/Au(111) surface calculated without (**a**) and with (**b**) spin–orbit interaction. The surface and quantum-well states and resonances are indicated by red dots. The states discussed in the text are labeled by symbols. In (**c**), the layer density of states is shown for the Pt, top three and central Au atomic layers. Blue and pink regions show the excess of LDOS in, respectively, Pt and Au layers in comparison with the corresponding bulk values.

**Figure 7:**
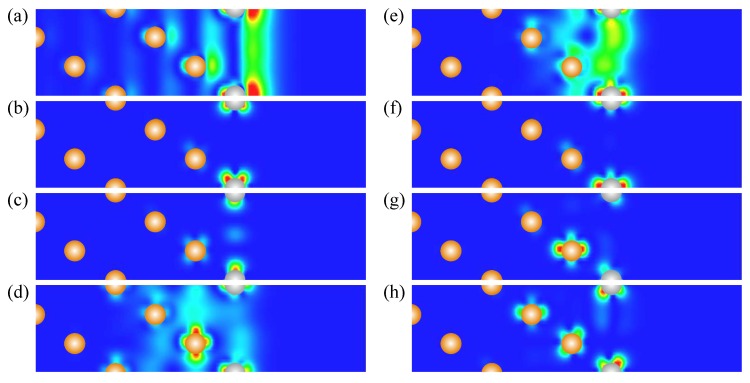
Charge density distribution of (a) $\Gamma_{1}$, (b) $\Gamma_{2}$, (c) $\Gamma_{4}$, (d) $M_{5}$, (e) $M_{6}$, (f) $K_{7}$, (g) $K_{8}$, (h) $K_{9}$ surface, quantum-well, and interface states in the 1 ML-Pt/Au(111) heterostructure. The Pt and Au atomic positions are shown by grey and yellow dots, respectively.

Figure 6 presents the electronic structure of the 1 ML Pt/Au(111) heterostructure. Its comparison with the electronic structure of clean Au(111) surface of Figure 4 shows that adsorption of one Pt ML leads to the dramatic changes in the electronic structure of the surface. In contrast to a pure Au(111) surface in the unoccupied part of the WSOC electronic structure of Figure 6a, there is a *s*-*p*-type surface state $\Gamma_{1}$ with energy of 0.23 eV at the $\bar{\Gamma }$ point. The charge density distribution of this state at $\bar{\Gamma }$ is reported in Figure 7. About 70% of this state is confined to the Pt atomic layer, i.e., this state resembles more a Pt surface state rather than that of the Au surface. Furthermore, such high level of confinement of this state to the surface atomic layer is larger than in the case of the surface state $\Gamma_{1}$ on the Pt(111) surface. This is confirmed by comparison of its charge density distribution in Figure 2 and Figure 7. As a result, one can conclude that deposition of one Pt ML on the Au(111) substrate results in complete disappearance of a partly occupied *s*-*p* surface state at the $\bar{\Gamma }$ point inherent of a clean Au(111) surface.

In the WSOC electronic structure of the 1 ML-Pt/(Au111) heterostructure shown in Figure 6a, in the vicinity of the $\bar{\Gamma }$ point in the energy interval studied here, in addition to the bulk-like electronic states of the Au(111) substrate, there are three almost totally occupied *d*-type energy bands generated by the Pt atoms. Two upper energy Pt-derived QWSs $\Gamma_{2}$ and $\Gamma_{3}$ meet each other at energy of −0.32 eV at $\bar{\Gamma }$. The dispersion of the upper $\Gamma_{2}$ band is almost flat. In the WSOC calculation, it conserves its true electronic state character almost over the whole $\bar{\Gamma }\;\bar{M}$ direction, whereas, along $\bar{\Gamma }\;\bar{K},$ it transforms into a resonance entering the projected bulk states continuum of the substrate

On the other hand, the $\Gamma_{3}$ band experiences a strong downward dispersion upon moving away from the $\bar{\Gamma }$ point. This state is a true electronic state in the wave-vector-energy region corresponding to the energy gap of the Au(111) substrate. Upon leaving this gap, it quickly transforms into a resonant state.

The lower energy state $\Gamma_{4}$ with energy of −2.0 eV at $\bar{\Gamma }$ has a rather flat dispersion in the vicinity of the SBZ center. However, at finite wave vectors, its energy starts to increase very quickly. Along the $\bar{\Gamma }\;\bar{K}$ direction, it almost reaches the Fermi level, whereas, along $\bar{\Gamma }\;\bar{K},$ it experiences avoiding-crossing interaction with the $\Gamma_{1}$ band at around −0.4 eV. After that, this band crosses the Fermi level and reaches the energy of ∼0.6 eV above the Fermi level at the $\bar{M}$ point.

The quantum-well character of these Pt-originated *d*-like states is confirmed from the analysis of its spatial localization. As seen in Figure 7, the charge density of the $\Gamma_{2}$ state is mainly confined to the Pt ML due to its *d* orbital character and effective reflection caused by the existence of the energy gaps in the electronic structure of the Au substrate and vacuum sides. Only a small fraction of its charge can be detected in the vicinity of Au atoms forming the first atomic layer of the substrate. The spatial localization of the $\Gamma_{4}$ state in the first Au atomic layer is larger as seen in Figure 7 due to hybridization with the Au *d* states. Nevertheless, its main portion resides around the Pt adatoms and does not penetrate into the substrate.

In the unoccupied part of the 1 ML-Pt/Au(111) WSOC electronic structure of Figure 6a, one can observe two bands with strong localization at the surface labeled as $M_{5}$ and $M_{6}$ located in the energy gap of Au(111) with energies of 1.4 and 2.4 eV at the $\bar{M}$ point. Charge density distribution of $M_{5}$ state presented in Figure 7 reveals that wave function of this state is almost equally distributed between the Pt and surface Au atomic layer. Significant density resides also in the second and third Au atomic layers. From such behavior, we interpret this state as a hybridized Au surface state strongly modified by Pt adsorption. On the contrary, the charge density of the $M_{6}$ state is strongly localized in the Pt atomic layer. It penetrates notably into the first Au atomic layer with only a small fraction in the second Au atomic layer. Such strong localization at the surface can be explained by localization of this state far inside the energy gap of the Au(111) substrate.

Additionally, the adsorbed Pt ML generate several electronic states in the wide energy gap in Au(111) around the $\bar{K}$ point strongly confined to the surface. In contrast to a clean Au(111) surface where no any surface state exists in the gap, in the WSOC 1 ML-Pt/Au(111) electronic structure of Figure 6a, we observe three energy bands with wave function strongly linked to the surface region. The upper energy state $K_{7}$ is a clear QWS generated by the Pt adlayer. This is confirmed by its strong spatial confinement to it as seen in Figure 7. As seen in Figure 7, the lower state $K_{8}$ is mainly localized in the surface Au atomic layer with significant part residing inside the Pt adlayer. The state $K_{9}$ is confined to the Pt and top two Au atomic layers.

When the spin–orbit interaction is taken into account, the dispersion of the surface and QWSs in 1 ML-Pt/Au(111) changes significantly as demonstrated in the comparison of Figure 6a,b. From the SOC surface electronic structure of Figure 6b, one can deduce that switching on the spin–orbit interaction leads to the lowering the energy position of the $\Gamma_{1}$ band. Thus, in Figure 6b, it has energy 0.1 eV above the Fermi level at the $\bar{\Gamma }$ point. Moreover, it experiences significant Bychkov–Rashba splitting into two spin-resolved bands with the splitting coefficient $\alpha_{R}$ = 1.5 eV·Å. This value is noticeably larger than the analogous splitting coefficient for the *s*-*p*-type state in Au(111) (0.4–0.8 eV·Å (see references [30,31,32] and present calculations). This is an intriguing result that can make this system attractive from the point of view of spintronics.

Figure 6b shows that the *d*-type $\Gamma_{2}$ and $\Gamma_{3}$ QWSs split into two separate sets of spin-resolved energy bands ($\Gamma_{2^{\prime }}$,$\Gamma_{2^{\prime \prime }}$) and ($\Gamma_{3^{\prime }}$,$\Gamma_{3^{\prime \prime }}$), respectively. The energy separation between these two sets of bands at $\bar{\Gamma }$ is about 0.6 eV in the SOC calculation and comparable with energy splitting of a double-degenerate band I along the ΓL direction of the bulk Brillouin zone of Figure 3b. Each pair of these bands is degenerate only at the $\bar{\Gamma }$ point and its dispersion has shape of a Dirac cone similar to that realized in the topological materials [101,102,103,104]. These four energy bands continue to be true electronic states inside the Au(111) *s*-*p* energy gap. However, leaving this energy gap, these QWS bands experience strong hybridization with the gold bulk-like electronic states. Moreover, in contrast to the WSOC case, the $\Gamma_{2^{\prime }}$ and $\Gamma_{3^{\prime }}$ QWSs efficiently transform into resonances outside the energy gap. The effect of spin–orbit interaction is even more dramatic on the $\Gamma_{2^{\prime \prime }}$ and $\Gamma_{3^{\prime \prime }}$ states, which quickly lose their surface-like character leaving the energy gap of the substrate.

We do not detect a noticeable effect of the spin–orbit interaction on the spin splitting of the $\Gamma_{4}$ QWS. Probably, this is related to its origin in the non-dispersing part of the band I along the ΓL direction, which does not experience significant spin–orbit splitting as seen in Figure 3b. On the other hand, its energy position shifts upward slightly, reaching −1.9 eV at the $\bar{\Gamma }$ point. Due to a strong hybridization of the upper $\Gamma_{2}$ band with gold bulk-like electronic states along the $\bar{\Gamma }\;\bar{M}$ direction, the $\Gamma_{4}$ band does not experience notable hybridization with that band in the SOC case and disperses as a very wide resonance at significantly lower energies towards the $\bar{M}$ point. Instead, along $\bar{\Gamma }\;\bar{K}$, its dispersion is hardly affected by the spin–orbit coupling.

In the calculated SOC electronic structure of 1 ML-Pt/Au(111) in Figure 6b, the spin–orbit interaction produces spin-spitting of $M_{5}$ and $M_{6}$ bands into two spin-resolved bands around the $\bar{M}$ point. At the same time, these spin-split bands cross each other at the $\bar{M}$ point (Bychkov–Rashba mechanism), although the magnitude of this splitting does not exceed 0.05 eV for these states.

Significant spin–orbit splitting can be observed in the QWS and interface states around the $\bar{K}$ point. As a result, the three spin-degenerate energy bands residing in the Au(111) energy gap at this point transform into six spin-resolved ones. The energy separation between the spin-split bands reaches 0.35 eV, as occuring between the $K_{7^{\prime }}$ and $K_{7^{\prime \prime }}$ bands along both the $\bar{K}\;\bar{M}$ and $\bar{K}\;\bar{\Gamma }$ symmetry directions. Moreover, since the shape of the lower edge of the Au bulk states continuum changes considerably by spin–orbit interaction, the dispersion of the lower energy bands notably deviate from the WSOC ones. For instance, the $K_{9}$ band in the WSOC calculation enters the projected bulk states continuum at the upper edge of the Au(111) energy gap. Instead, the $K_{9^{\prime }}$ and $K_{9^{\prime \prime }}$ bands in the SOC electronic structure originated from this band cross the lower edge of that energy gap.

Deposition of a Pt ML on the Au(111) substrate produces strong modifications in the LDOS as shown in Figure 6c in comparison with both the Pt(111) (Figure 1c) and Au(111) (Figure 4c) clean surfaces. One can see that the LDOS for the adsorbed Pt ML at the Fermi level is about 30% lower than that for a pure Pt(111) surface. This can be explained by the lack at the Fermi level of *d*-type electronic states in the Pt adlayer. Even the upward shift of the $\Gamma_{2^{\prime }}$ and $\Gamma_{2^{\prime \prime }}$ QWSs upon switching on the spin–orbit interaction increases LDOS at the Fermi level by 10% only. Instead, we observe strong enhancement in the Pt ML LDOS in the −0.3 ÷−1.8 eV energy interval. This is explained by the presence of the Pt *d*-like states in this energy region at $\bar{\Gamma }$ and $\bar{K}$ quantified due to a spatial confinement in the direction perpendicular to the surface. Since the same states spatially penetrate into the surface and subsurface Au atomic layers, a notable increase of LDOS of those layers in the same energy region is observed in Figure 6c as well. Thus, the LDOS in the first Au layer is drastically enhanced at these energies in comparison with that of any atomic layer of the clean Au(111) surface of Figure 4c, as well as from deeper layers of the heterostructure. Even in the second Au atomic layer, the LDOS at −1.25 eV is enhanced twice due to contribution from the Pt QWSs.

#### 3.3.2. 2 ML-Pt/Au(111)

**Figure 8:**
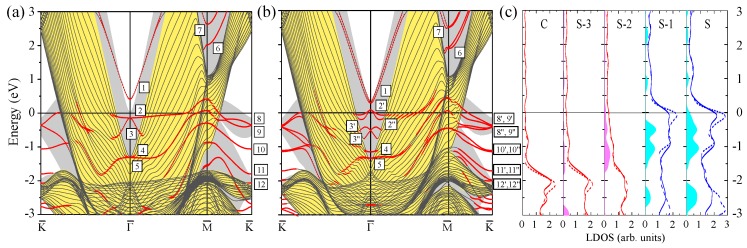
The electronic structure of the 2 ML-Pt/Au(111) surface calculated without (**a**) and with (**b**) spin–orbit interaction. The surface and quantum-well states and resonances are indicated by red dots. The states discussed in the text are labeled by symbols. In (**c**), the layer density of states is shown for two Pt, top two and central Au atomic layers. Blue and pink regions show the excess of LDOS in, respectively, Pt and Au layers in comparison with the corresponding bulk values.

**Figure 9:**
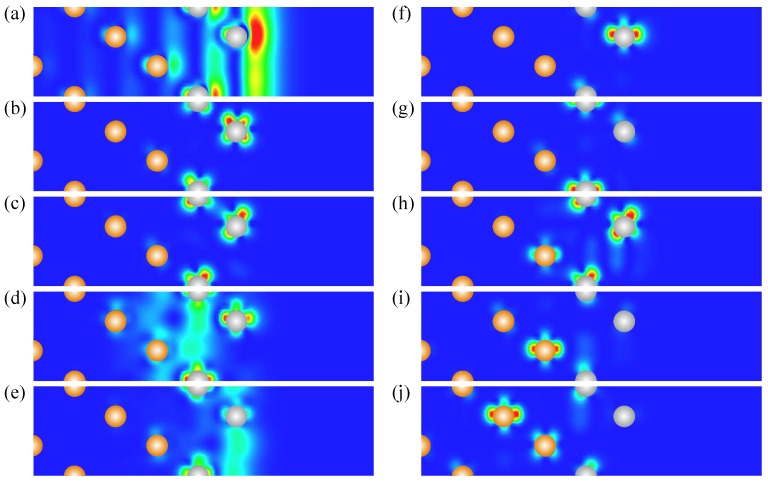
Charge density distribution of (a) $\Gamma_{1}$, (b) $\Gamma_{2}$, (c) $\Gamma_{4}$, (d) $M_{6}$, (e) $M_{7}$, (f) $K_{8}$, (g) $K_{9}$, (h) $K_{10}$, (i) $K_{11}$, (j) $K_{12}$ surface, quantum-well, and interface states in the 2 ML-Pt/Au(111) heterostructure. The Pt and Au atomic positions are shown by grey and yellow dots, respectively.

The electronic structure of 2 ML-Pt/Au(111) presented in Figure 8 demonstrates that deposition of additional Pt atomic layer results in strong variations in almost all regions. As in the previous 1 ML-Pt/Au(111) case in the energy gap at $\bar{\Gamma }$, we observe the *s*-*p*-type surface state $\Gamma_{1}$. Increase of the Pt adsorbate thickness produces its slight shift upward. Thus, in the WSOC case of Figure 8a, this state is located at 0.4 eV above the Fermi level at $\bar{\Gamma }$. As in the case of one Pt adlayer, it disperses upward and keeps its surface character even at larger energies. The charge density distribution of this state shown in Figure 9 reveals its significant penetration into the second Pt atomic layer. Some part of this state can be detected in the vicinity of the first and second Au atomic layers as well. Nevertheless, the penetration of this state into the gold substrate is significantly reduced in comparison with the 1 ML-Pt/Au(111) case.

Below the Fermi level in the WSOC electronic structure, we observe three *d*-like energy bands strongly localized in the Pt atomic layers. The dispersion of the $\Gamma_{2}$ and $\Gamma_{3}$ bands in Figure 8a is qualitatively similar to that given in Figure 6a. However, in the 2 ML-Pt/Au(111) system, these bands are located significantly closer to the Fermi level. For instance, they meet each other at the $\bar{\Gamma }$ point at energy only of −0.13 eV. Moreover, because of its almost flat dispersion, the $\Gamma_{2}$ QWS band disperses almost parallel to the Fermi level, which drastically increases the LDOS in vicinity of the Fermi level, as will be discussed below. Being located at higher energy in the energy gap of the Au(111) substrate, these states experience stronger repulsion from the Au(111) energy gap as well, which result in its stronger confinement to the Pt adlayer.

As seen in Figure 8a, the increase of the Pt film thickness generates the $\Gamma_{4}$ and $\Gamma_{5}$ QWS bands, which are degenerated at $\bar{\Gamma }$ meeting at −1.3 eV. Due to such energy position, they have their origin in the strongly dispersing part of the bulk band I along the ΓL direction presented in Figure 3b. The charge density distribution of the state $\Gamma_{4}$ shown in Figure 9 confirms its strong spatial confinement to the top Pt two atomic layers and orbital composition similar to that of the $\Gamma_{2}$ and $\Gamma_{3}$ states. The upper energy $\Gamma_{4}$ band has positive dispersion and quickly loses its surface character moving away from the $\bar{\Gamma }$ point. Instead, the $\Gamma_{5}$ band propagates over a much more extended wave vector region. Along the $\bar{\Gamma }\;\bar{M}$ direction, this band initially is almost dispersionless. At wave vectors exceeding the 4/10 distance between the $\bar{\Gamma }$ and $\bar{M}$ points, this band begins to disperse rather steeply towards the Fermi level, reaching it around the $\bar{M}$ point. Instead, in the $\bar{\Gamma }\;\bar{K}$ direction, the $\Gamma_{5}$ band has initial negative dispersion and drops down to energy of −1.8 eV. After that, its dispersion turns out to be positive. At energies above −0.9 eV, this state converts into a weak resonance caused by its interaction with the Pt-derived $\Gamma_{3}$ QWS and bulk Au states.

In the Au(111) energy gap above the Fermi level at the $\bar{M}$ point, the number of states linked to the surface is maintained to be two. However, in contrast to the 1 ML-Pt/Au(111) heterostructure, the $M_{6}$ and $M_{7}$ bands in 2 ML-Pt/Au(111) are located at significantly higher energies. As a result, the $M_{6}$ state strongly reduces its penetration into the gold substrate as seen in Figure 9. In the same figure, one can see how the charge density of the $M_{7}$ state at $\bar{M}$ almost entirely localizes in the two Pt atomic layers. Only its small fraction penetrates into the first Au layer.

At the $\bar{K}$ point, we also observe the increase in the number of states linked to the Pt adlayer. Now, we can detect up to five such bands in the upper energy gap of Au(111). In close vicinity of the Fermi level, there are two bands labeled as $K_{8}$ and $K_{9}$ dispersing rather closely to each other and meeting at the $\bar{K}$ point at −0.34 eV. Note that these bands are located in the grey region showing the bulk projected states continuum of Pt(111) signalling about their clear QWS character. This is confirmed by the spatial localization of the $K_{8}$ and $K_{9}$ states almost entirely into the surface and second Pt layers, respectively, as seen in Figure 9.

Instead, dispersion of the lower energy band $K_{10}$ partly occurs in the energy gap of both Pt and Au. Despite this, Figure 9 shows that its wave function at $\bar{K}$ is fairly equally distributed between two Pt layers. The $K_{11}$ state is located in both Pt and top Au atomic layers. Figure 9 demonstrates its hybridized character and reveals that, in the vicinity of Pt atoms, it has a clear $d_{xy},d_{xz},d_{yz},d_{x^{2}-y^{2}}$ character, whereas, around the Au atoms, it possesses the $d_{3z^{2}-r^{2}}$ symmetry.

As it occurs in the 1 ML-Pt/Au(111) heterostructure, the spin–orbit interaction strongly modifies the electronic structure of the 2 ML-Pt/Au(111) system, as one can deduce comparing Figure 8a,b. Again, the $\Gamma_{1}$ surface state spits into two spin-resolved bands with $\alpha_{R}$ = 1.6 eV·Å and shifts slightly downward. It is located at 0.3 eV above the Fermi level at the $\bar{\Gamma }$ point.

Impact of the spin–orbit interaction on the electronic structure at the Fermi level is especially dramatic in the 2 ML-Pt/Au(111) system. Its inclusion places the $\Gamma_{2^{\prime }}$ and $\Gamma_{2^{\prime \prime }}$ QWS bands exactly at the Fermi level. Interestingly, in the SOC case, these and the $\Gamma_{3^{\prime }}$ and $\Gamma_{3^{\prime \prime }}$ spin-resolved bands have a clear surface character only inside the Au(111) energy gap forming the Dirac cones. Leaving this gap, they quickly transform into weak resonances.

Spin-orbit interaction produces the splitting of the lower energy bands $\Gamma_{4}$ and $\Gamma_{5}$ as well. Although the energy separation between bands $\Gamma_{4}$ and $\Gamma_{5}$ at $\bar{\Gamma }$ is notably smaller, the upper energy band $\Gamma_{4}$ shifted upward maintains its surface-like character inside the energy gap of the substrate. Reaching the edges of this gap, it quickly transforms into a weak resonance. In contrast to the above surface and QWS bands, we find that this band is spin-degenerate. The same occurs with the lower energy band $\Gamma_{5}$, which, even in the SOC case, is spin-degenerate at all wave vectors.

In the case of the 2 ML-Pt/Au(111) system, the spin–orbit coupling produces some effect on the surface and QWS bands in the vicinity of the $\bar{M}$ and $\bar{K}$ points. The strength of its effect on dispersion and spin–orbit splitting of these energy bands is similar to that discussed in the case of the 1 ML-Pt/Au(111) system. However, the important difference is that the spin–orbit splitting strongly modifies the dispersion of the upper energy bands $K_{8^{\prime }}$ and $K_{9^{\prime }}$ around the $\bar{K}$ point, pushing them up towards the Fermi level like it occurs in the case of the upper QWSs at the $\bar{\Gamma }$ point.

Presence of the Pt-derived QWSs in the vicinity of the Fermi level strongly enhances the LDOS in the surface Pt layer. As seen in Figure 8c, the LDOS in the surface Pt layer exceeds by about 20% its bulk value in Pt. This is accompanied by strong enhancement of the LDOS in the surface layer in a energy window from −1.67 to 0.2 eV. The excess of charge in the surface layer can be observed at energies below −2 eV as well. The LDOS in the second Pt layer also significantly exceeds the bulk Pt values in wide energy intervals. The largest enhancement is observed in the energy interval from −1.64 to −0.2 eV. Interestingly, in fairly the same energy interval, a notable increase of LDOS in the first Au atomic layer presents as well, which can be explained by a spatial penetration of the Pt-derived QWS into the gold substrate.

#### 3.3.3. 3 ML-Pt/Au(111)

**Figure 10:**
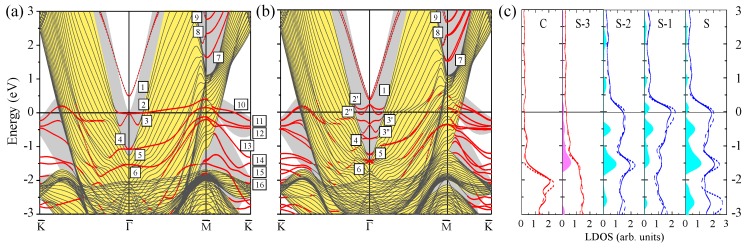
The electronic structure of the 3 ML-Pt/Au(111) surface calculated without (**a**) and with (**b**) spin–orbit interaction. The surface and quantum-well states and resonances are indicated by red dots. The states discussed in the text are labeled by symbols. In (**c**), the layer density of states is shown for three Pt, top and central Au atomic layers. Blue and pink regions show the excess of LDOS in, respectively, Pt and Au layers in comparison with the corresponding bulk values.

**Figure 11:**
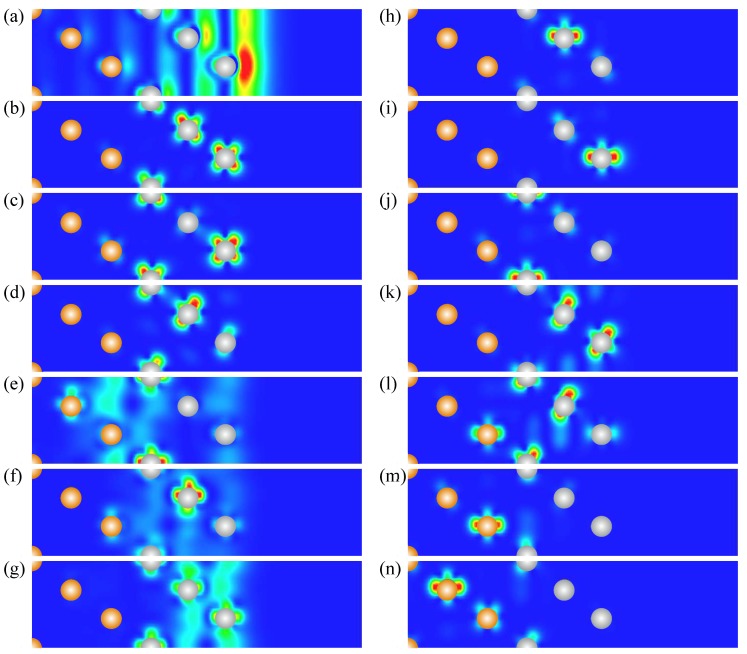
Charge density distribution of (a) $\Gamma_{1}$, (b) $\Gamma_{2}$, (c) $\Gamma_{4}$, (d) $\Gamma_{6}$, (e) $M_{7}$, (f) $M_{8}$, (g) $M_{9}$, (h) $K_{10}$, (i) $K_{11}$, (j) $K_{12}$, (k) $K_{13}$, (l) $K_{14}$, (m) $K_{15}$, (n) $K_{16}$ surface, quantum-well, and interface states in the 3 ML-Pt/Au(111) heterostructure. The Pt and Au atomic positions are shown by grey and yellow dots, respectively.

An increasing number of adsorbed Pt atomic layers up to three has little effect on the energy dispersion of the $\Gamma_{1}$ surface state, as can be seen in Figure 10. In the WSOC case, it is located in respect to the Fermi level in a position similar to that in the 2 ML-Pt/Au(111) system. Its charge density distribution reported in Figure 11 reveals that it is very similar to that of a clean Pt(111) surface of Figure 1c. Only a small fraction of this state resides in the first and second Au atomic layers. Nevertheless, it seems such penetration into the gold substrate still has some effect on the energy position of this surface state since, at the $\bar{\Gamma }$ point, it is located about 0.2 eV higher energy than occurring on the clean Pt(111) surface.

Analyzing the WSOC electronic structure of 3 ML-Pt/Au(111) reported in Figure 10a, one can notice an increasing number of Pt-derived states in all three Au(111) energy gaps of interest here. Thus, around the $\bar{\Gamma },$ we observe five QWS bands. The upper four of them are located in the Au(111) energy gap and are true QWSs. On the contrary, the lower energy $\Gamma_{6}$ state at all wave vectors coexist with the Au bulk-like states. Nevertheless, due to the difference of its symmetry with that of the *s*-*p* electronic states forming the Au bulk continuum, the state $\Gamma_{6}$ is a well defined quantum state over a large region in the SBZ in the WSOC calculation. The charge density distributions of the $\Gamma_{2}$, $\Gamma_{4}$ and $\Gamma_{6}$ states reported in Figure 11 confirm its *d*-like character and confinement to the Pt film. Thus, one can see that the $\Gamma_{2}$ state is mainly localized in all three of the Pt atomic layers, having maximal value at the surface atoms. On the contrary, the $\Gamma_{4}$ state has two similar maxima on the first and third Pt atomic layer and is negligible in the vicinity of the second Pt layer. Wave function of $\Gamma_{6}$ state has a maximum on the second Pt layer, whereas its amplitude on the surface layer is relatively small.

At the $\bar{M}$ point in the upper energy gap of the gold substrate in the electronic structure of the 3 ML-Pt/Au(111) system, we observe three states labeled as $M_{7}$, $M_{8}$ and $M_{9}$. Analysis of a charge density distribution of the $M_{7}$ reported in Figure 11 reveals that it resides mainly in the Pt and Au atomic layers forming the interface. On the other hand, the charge density distribution plots presented in Figure 11 demonstrate that the $M_{8}$ and $M_{9}$ are typical QWSs, since they are strongly confined to the Pt adlayer.

In the electronic structure of Figure 10a, one can observe three energy bands labeled $K_{10}$, $K_{11}$, and $K_{12}$, which correlates with the presence of three atomic layers in the Pt film. Moreover, the upper energy band $K_{10}$ crosses the Fermi level in both symmetry directions, whereas the $K_{11}$ and $K_{12}$ states are totally occupied being located inside the Au(111) energy gap. Analysis of the charge density distributions of these three states at the $\bar{K}$ point reported in Figure 11 reveals an interesting situation that contradicts a conventional particle-in-box model. The upper energy state $K_{10}$ is almost entirely localized in the second Pt atomic layer. The $K_{11}$-state wave function resides mostly in Pt atoms forming the surface layer. Finally, the $K_{12}$ state has strong localization in the third Pt atomic layer. We explain such strong localization of these QWSs in certain atomic layers by the almost flat dispersion of the upper energy bulk band generating (presented in Figure 3c) these QWSs.

In the lower part of the Au(111) energy gap, we observe three states reaching the $\bar{K}$ point. The charge density distribution in Figure 11 shows that the upper energy $K_{13}$ state has localization in the top two Pt atomic layers. The wave function of the $K_{14}$ state spreads over the second and third Pt and first Au atomic layers. On the contrary, the $K_{15}$ state localizes in the first Au atomic layer with only a small portion residing around the Pt atoms in the third layer. The $K_{16}$ localizes at the Pt/Au interface with the maximum in the second Au atomic layer.

As in the previous heterostuctures, the spin–orbit interaction produces a strong impact on the electronic structure of the 3 ML-Pt/Au(111) system. As seen in Figure 10b above the Fermi level, the surface, quantum-well and interface *s*-*p*-like states at the $\bar{\Gamma }$ and $\bar{M}$ points split into a couple of two spin-resolved energy bands due to the Bychkov–Rashba splitting mechanism. In particular, $\alpha_{R}$ is of 1.7 eV·Å for the $\Gamma_{1}$ state. Note that the region around the $\bar{\Gamma }$ point, where the fitting of the $\Gamma_{1}$ surface state dispersion in the Bychkov–Rashba model is valid, reduces upon increase of the Pt adlayer thickness due to hybridization with the bulk-like QWSs. Eventually, at sufficiently thick Pt film, the determination of $\alpha_{R}$ will be impossible.

In the Au(111) energy gap in the vicinity and below the Fermi level at the $\bar{\Gamma }$ point, the Pt-derived QWSs experience the spin–orbit splitting in the same manner as it occurs in the heterostructures with one and two adsorbed Pt atomic layers. The magnitude of spin-splitting is at a maximum in the $\Gamma_{2}$, $\Gamma_{3}$, $\Gamma_{4}$ and $\Gamma_{5}$ QWSs located at the higher energies and is minimal in the $\Gamma_{6}$ QWS band located at lower energy. One can notice that the energy splitting among the ($\Gamma_{2^{\prime }}$, $\Gamma_{2^{\prime \prime }}$) and ($\Gamma_{3^{\prime }}$, $\Gamma_{3^{\prime \prime }}$) bands at the $\bar{\Gamma }$ point is fairly the same as in the previous systems with Pt adlayers, in such a way confirming its origin in the bulk bands I${}^{\prime }$ and I${}^{\prime \prime }$ along the ΓL direction of Figure 3b. As a result, the $\Gamma_{2^{\prime }}$ state becomes completely unoccupied in the 3 ML-Pt/Au(111) system, whereas the $\Gamma_{2^{\prime \prime }}$ crosses the Fermi level two times in each symmetry direction.

As seen in Figure 10b, six spin-degenerate energy bands located in the Au(111) energy gap at $\bar{K}$ in the WSOC electronic structure of 3 ML-Pt/Au(111) transforms into twelve spin-resolved bands upon switching on the spin–orbit interaction. Comparison with the SOC electronic structure in systems with one and two Pt atomic layers shows that the spin–orbit splitting is notably smaller in a system containing three Pt layers. On the other hand, from such a comparison, one can deduce that the spin–orbit splitting of the states at the $\bar{K}$ point is maximal in 1 ML-Pt/Au(111) and gradually reduces with the increase of atomic layers in the Pt film. Eventually, it disappears in thick Pt films.

The calculated LDOS of 3 ML-Pt/Au(111) shown in Figure 10c presents differences with that of the clean Pt(111) surface. Thus, in the surface Pt layer, we find the extra charge just above the Fermi level caused by the presence of the $\Gamma_{2}$ QWS. The shape of the LDOS for this atomic layer below the Fermi level is distorted in comparison with that of Figure 1. However, the main modifications in the LDOS occur in the second and third Pt atomic layer. Thus, comparing the LDOS in Figure 1c and Figure 10c, we observe accumulation of the extra charge in these layers in the 3 ML-Pt/Au(111) heterostucture. Note also the accumulation of charge in the interface Au layer at and below the Fermi level in this system.

Analyzing the LDOS in all the systems studied, we observe that, in comparison with the clean Pt(111) surface, the strongest enhancement in the surface atomic layer is observed in the 1 ML-Pt/Au(111) heterostructure. To be specific, the LDOS integrated in the −3 eV to 0 interval exceeds that on the clean Pt surface by about 20%. According to the model proposed in Ref. [18], such increase would result in increasing reactivity of the Pt/Au(111) systems. In 2 ML-Pt/Au(111), this value drops to 8% and reduces to 7% in 3 ML-Pt/Au(111). This tendency correlates with the experimental observation of maximal catalytic activity in a 1 ML Pt film on the gold surface. This activity decreases monotonically with the increase of film thickness and reaches its bulk Pt behavior beyond three Pt MLs [27].

## 4. Conclusions

We studied the electronic structure of the atomically thin Pt films deposited on the Au(111) surface in the framework of the density-functional theory. The calculated electronic structure of the n ML-Pt/Au(111) heterostructures containing one, two and three Pt atomic layers allowed us to investigate in detail the formation of Pt-derived quantum-well and interface states upon variation of the number of atomic layers. In particular, we scrutinized the evolution of the Pt-induced states in wide energy gaps of Au(111) in vicinity of the $\bar{\Gamma }$, $\bar{M}$, and $\bar{K}$ points in the SBZ.

We show that the *s*-*p* Shockley surface state of Au(111) becomes totally unoccupied in all Pt-covered systems studied. The deposition of the Pt adlayers results in the appearance of the *d*-type spin-resolved QWSs in the wide energy gap at the SBZ center. Comparison of its spatial localization in the systems with different Pt adsorbate thickness confirms its a particle-in-box scenario. The spin–orbit splitting of these states is unusually large. In a significant energy range, the dispersion of these spin-resolved QWS bands presents a Dirac-cone-like shape.

Above the Fermi level at the $\bar{M}$ point of the SBZ, we observe formation of *s*-*p*-like Pt-derived QWSs and transformation of the Au(111) surface state into the Pt/Au interface state. Around the $\bar{K}$, the Pt adlayer generates *d*-type QWSs with strong localization at the surface. Analysis of its spatial localization reveals that each such quantum state localizes almost entirely in a certain Pt atomic layer, contradicting a particle-in-box model. We explain such behavior by almost flat dispersion of the bulk energy band that produces these QWSs.

The presence of a large number of the strongly localized surface, quantum-well and interface states results in strong enhancement of the LDOS in the n ML-Pt/Au(111) systems in comparison with that of the clean Pt(111) and Au(111) surfaces. Such enhancement correlates with the increasing catalytic activity of such systems. 

## Abbreviations

The following abbreviations are used in this manuscript:

ML	monolayer
QWS	quantum-well state
LDOS	layer density of states
SBZ	surface Brillouin zone
SOC	spin–orbit coupling
WSOC	without spin–orbit coupling

## References

[1] Nielsen L.P., Besenbacher F., Stensgaard I., Lægsgaard E. (1995). Dealloying phase-separation during growth of Au on Ni(110). Phys. Rev. Lett..

[2] Meyer J.A., Baikie I.D., Kopatzki E., Behm R.J. (1996). Preferential island nucleation at the elbows of the Au(111) herringbone reconstruction through place exchange. Surf. Sci..

[3] Shern C.S., Tsay J.S., Fu T.Y. (1996). Growth mechanism of Ni on Pt(110) at low temperature. Appl. Surf. Sci..

[4] Steltenpohl A., Memmel N., Taglauer E., Fauster T., Onsgaard J. (1997). Pd, Au and codeposited Pd-Au ultrathin films on Ru(001). Surf. Sci..

[5] Bertolinn J.C. (2000). Surface stress and chemical reactivity of Pt and Pd overlayers. Appl. Catal. A.

[6] Maroun F., Ozanam F., Magnussen O.M., Behm R.J. (2001). The role of atomic ensembles in the reactivity of bimetallic electrocatalysts. Science.

[7] Waibel H.F., Kleinert M., Kibler L.A., Kolb D.M. (2002). Initial stages of Pt deposition on Au(111) and Au(100). Electrochim. Acta.

[8] Mugarza A., Mascaraque A., Repain V., Rousset S., Altmann K.N., Himpsel F.J., Koroteev Y.M., Chulkov E.V., de Abajo F.J.G., Ortega J.E. (2002). Lateral quantum wells at vicinal Au(111) studied with angle-resolved photoemission. Phys. Rev. B.

[9] Okada M., Nakamura M., Moritani K., Kasai T. (2003). Dissociative adsorption of hydrogen on thin Au films grown on Ir111. Surf. Sci..

[10] Morgenstern K., Kibsgaard J., Lauritsen J.V., Lægsgaard E., Besenbacher F. (2007). Cobalt growth on two related close-packed noble metal surfaces. Surf. Sci..

[11] Allmers T., Donath M. (2009). Growth and morphology of thin Fe films on flat and vicinal Au(111): A comparative study. New J. Phys..

[12] Baber A.E., Tierney H.L., Sykes E.C.H. (2010). Atomic-scale Geometry and electronic structure of catalytically important Pd/Au alloys. ACS Nano.

[13] Eyrich M., Diemant T., Hartmann H., Bansmann J., Behm R.J. (2012). Interaction of CO with structurally well-defined monolayer PtAu/Pt(111) surface alloys. J. Phys. Chem. C.

[14] Krupski K., Moors M., Jozwik P., Kobiela T., Krupski A. (2015). Structure determination of Au on Pt(111) surface: LEED, STM and DFT study. Materials.

[15] Chiang T.-C. (2000). Photoemission studies of quantum well states in thin films. Surf. Sci. Rep..

[16] Chulkov E.V., Borisov A.G., Gauyacq J.P., Sanchez-Portal D., Silkin V.M., Zhukov V.P., Echenique P.M. (2006). Electronic excitations in metals and at metal surfaces. Chem. Rev..

[17] Uosaki K., Ye S., Naohara H., Oda Y., Haba T., Kondo T. (1997). Electrochemical epitaxial growth of a Pt(111) phase on an Au(111) electrode. J. Phys. Chem. B.

[18] Pedersen M.O., Helveg S., Ruban A., Stensgaard I., Lægsgaard E., Nørskov J.K., Besenbahcer F. (1999). How a gold substrate can increase the reactivity of a Pt overlayer. Surf. Sci..

[19] Lou Y.B., Maye M.M., Han L., Luo J., Zhong C.J. (2001). Gold-platinum alloy nanoparticle assembly as catalyst for methanol electrooxidation. Chem. Commun..

[20] Du B.C., Tong Y.Y. (2005). A coverage-dependent study of Pt spontaneously deposited onto Au and Ru surfaces: Direct experimental evidence of the ensemble effect for methanol electro-oxidation on Pt. J. Phys. Chem. B.

[21] Kim J., Jung C., Rhee C.K., Lim T. (2007). Electrocatalytic oxidation of formic acid and methanol on Pt deposits on Au(111). Langmuir.

[22] Gohda Y., Groß A. (2007). Structure-reactivity relationship for bimetallic electrodes: Pt overlayers and PtAu surface alloys on Au(111). J. Electroanal. Chem..

[23] Gohda Y., Groß A. (2007). Local reactivity of ultrathin platinum overlayers and surface alloys on a gold surface. Surf. Sci..

[24] Jia J.B., Cao L.Y., Wang Z.H. (2008). Platinum-coated gold nanoporous film surface: Electrodeposition and enhanced electrocatalytic activity for methanol oxidation. Langmuir.

[25] Khosravi M., Amini M.K. (2010). Carbon paper supported Pt/Au catalysts prepared via Cu underpotential deposition-redox replacement and investigation of their electrocatalytic activity for methanol oxidation and oxygen reduction reactions. Int. J. Hydrog. Energy.

[26] Li M., Liu P., Adzic R.R. (2012). Platinum monolayer electrocatalysts for anodic oxidation of alcohols. J. Phys. Chem. Lett..

[27] Ahn S.H., Liu Y., Moffat T.P. (2015). Ultrathin platinum films for methanol and formic acid oxidation: Activity as a function of film thickness and coverage. ACS Catal..

[28] Peng L.Y., Gan L., Wei Y.P., Yang H., Li J., Du H.D., Kang F.Y. (2016). Pt submonolayers on Au nanoparticles: Coverage-dependent atomic structures and electrocatalytic stability on methanol oxidation. J. Phys. Chem. C.

[29] Bychkov Y.A., Rashba E.I. (1984). Properties of a 2D electron-gas with lifted spectral degeneracy. JETP Lett..

[30] Lashell S., McDougall B., Jensen E. (1996). Spin splitting of an Au(111) surface state band observed with angle resolved photoelectron spectroscopy. Phys. Rev. Lett..

[31] Reinert F., Nicolay G., Schmidt S., Ehm D., Hüfner S. (2001). Direct measurements of the L-gap surface states on the (111) face of noble metals by photoelectron spectroscopy. Phys. Rev. B.

[32] Reinert F. (2003). Spin-orbit interaction in the photoemission spectra of noble metal surface states. Phys. J. Condens. Matter.

[33] Hoesch M., Muntwiler M., Petrov V.N., Hengsberger M., Patthey L., Shi M., Falub M., Greber T., Osterwalder J. (2004). Spin structure of the Shockley surface state on Au(111). Phys. Rev. B.

[34] Mazzarello R., Dal Corso A., Tosatti E. (2008). Spin-orbit modifications and splittings of deep surface states on clean Au(111). Surf. Sci..

[35] Bentmann H., Abdelouahed S., Mulazzi M., Henk J., Reinert F. (2012). Direct observation of interband spin-orbit coupling in a two-dimensional electron system. Phys. Rev. Lett..

[36] El-Kareh L., Bihlmayer G., Buchter A., Bentmann H., Blügel S., Reinert F., Bode M. (2014). A combined experimental and theoretical study of Rashba-split surface states on the (root 3 × root 3) Pb/Ag (111)R30 degrees surface. New J. Phys..

[37] Yan B., Stadtmüller B., Haag N., Jakobs S., Seidel J., Jungkenn D., Mathias S., Cinchetti M., Aeschlimann M., Felser C. (2015). Topological states on the gold surface. Nat. Commun..

[38] Dal Corso A. (2015). Clean Ir(111) and Pt(111) electronic surface states: A first-principle fully relativistic investigation. Surf. Sci..

[39] Krasovskii E.E. (2015). Spin-orbit coupling at surfaces and 2D materials. J. Phys. Condens. Matter.

[40] Hirahara T., Nagao T., Matsuda I., Bihlmayer G., Chulkov E.V., Koroteev Y.M., Hasegawa S. (2007). Quantum well states in ultrathin Bi films: Angle-resolved photoemission spectroscopy and first-principles calculations study. Phys. Rev. B.

[41] Ast C.R., Henk J., Ernst A., Moreschini L., Falub M.C., Pacile D., Bruno P., Kern K., Grioni M. (2007). Giant spin splitting through surface alloying. Phys. Rev. Lett..

[42] Bihlmayer G., Blügel S., Chulkov E.V. (2007). Enhanced Rashba spin-orbit splitting in Bi/Ag(111) and Pb/Ag(111) surface alloys from first principles. Phys. Rev. B.

[43] Mathias S., Ruffing A., Deicke F., Wiesenmayer M., Sakar I., Bihlmayer G., Chulkov E.V., Koroteev Y.M., Echenique P.M., Bauer M. (2010). Quantum-well-induced giant spin-orbit splitting. Phys. Rev. Lett..

[44] Lindgren S.Å., Walldén L. (1987). Discrete valence-electron states in thin metal overlayers on a metal. Phys. Rev. Lett..

[45] Miller T., Samsavar A., Franklin G.E., Chiang T.-C. (1988). Quantum-well states in a metallic system: Ag on Au(111). Phys. Rev. Lett..

[46] Paggel J.J., Miller T., Chiang T.-C. (1999). Quantum-well states as Fabry-Perot modes in a thin-film electron interferometer. Science.

[47] Torsti T., Lindberg V., Puska M.J., Hellsing B. (2002). Model study of adsorbed metallic quantum dots: Na on Cu(111). Phys. Rev. B.

[48] Milun M., Pervan P., Woodruff D.P. (2002). Quantum well structures in thin metal films: Simple model physics in reality?. Rep. Prog. Phys..

[49] Guo Y., Zhang Y.F., Bao X.Y., Han T.Z., Tang Z., Zhang L.X., Zhu W.G., Wang E.G., Niu Q., Qiu Z.Q. (2004). Superconductivity modulated by quantum size effects. Science.

[50] Upton M.H., Wei C.M., Chou M.Y., Miller T., Chiang T.C. (2004). Thermal stability and electronic structure of atomically uniform Pb films on Si(111). Phys. Rev. B.

[51] Zhang Y.F., Jia J.F., Han T.Z., Tang Z., Shen Q.T., Guo Y., Qiu Z.Q., Xue Q.K. (2005). Band structure and oscillatory electron-phonon coupling of Pb thin films determined by atomic-layer-resolved quantum-well states. Phys. Rev. Lett..

[52] Corriol C., Silkin V.M., Sanchez-Portal D., Arnau A., Chulkov E.V., Echenique P.M., von Hofe T., Kliewer J., Kroger J., Berndt R. (2005). Role of elastic scattering in electron dynamics at ordered alkali overlayers on Cu(111). Phys. Rev. Lett..

[53] Dil J.H., Kim J.W., Kampen T., Horn K., Ettema A.R.H.F. (2006). Electron localization in metallic quantum wells: Pb versus In on Si(111). Phys. Rev. B.

[54] Lindberg V., Petersson T., Hellsing B. (2006). Quantum size effects of CO reactivity on metallic quantum dots. Surf. Sci..

[55] Kirchmann P.S., Wolf M., Dil J.H., Horn K., Bovensiepen U. (2007). Quantum size effects in Pb/Si(111) investigated by laser-induced photoemission. Phys. Rev. B.

[56] Breitholtz M., Chis V., Hellsing B., Lindgren S.A., Wallden L. (2007). Overlayer resonance and quantum well state of Cs/Cu(111) studied with angle-resolved photoemission, LEED, and first-principles calculations. Phys. Rev. B.

[57] Hong I.-P., Brun C., Patthey F., Sklyadneva I.Y., Zubizarreta X., Heid R., Silkin V.M., Echenique P.M., Bohnen K.P., Chulkov E.V., Schneider W.-D. (2009). Decay mechanisms of excited electrons in quantum-well states of ultrathin Pb islands grown on Si(111): Scanning tunneling spectroscopy and theory. Phys. Rev. B.

[58] Qin S., Kim J., Niu Q., Shih C.K. (2009). Superconductivity at the two-dimensional limit. Science.

[59] Zugarramurdi A., Zabala N., Silkin V.M., Borisov A.G., Chulkov E.V. (2009). Lifetimes of quantum well states and resonances in Pb overlayers on Cu(111). Phys. Rev. B.

[60] Brun C., Hong I.P., Patthey F., Sklyadneva I.Y., Heid R., Echenique P.M., Bohnen K.P., Chulkov E.V., Schneider W.D. (2009). Reduction of the Superconducting Gap of Ultrathin Pb Islands Grown on Si(111). Phys. Rev. Lett..

[61] Zhang T., Cheng P., Li W.J., Sun Y.J., Wang G., Zhu X.G., He K., Wang L., Ma X., Chen X. (2010). Superconductivity in one-atomic-layer metal films grown on Si(111). Nat. Phys..

[62] Kirchmann P.S., Rettig L., Zubizarreta X., Silkin V.M., Chulkov E.V., Bovensiepen U. (2010). Quasiparticle lifetimes in metallic quantum-well nanostructures. Nat. Phys..

[63] Han Y., Ünal B., Jing D., Thieal P.A., Evans J.W., Liu D.-J. (2010). Nanoscale “Quantum” islands on metal substrates: Microscopy studies and electronic structure analyses. Materials.

[64] Rettig L., Kirchmann P.S., Bovensiepen U. (2012). Ultrafast dynamics of occupied quantum well states in Pb/Si(111). New J. Phys..

[65] Zugarramurdi A., Zabala N., Silkin V.M., Chulkov E.V., Borisov A.G. (2012). Quantum-well states with image state character for Pb overlayers on Cu(111). Phys. Rev. B.

[66] Slomski B., Landolt G., Muff S., Meier F., Osterwalder J., Dil J.H. (2013). Interband spin-orbit coupling between anti-parallel spin states in Pb quantum well states. New J. Phys..

[67] Fan Y.T., Lo M.C., Wu C.C., Chen P.Y., Wu J.S., Liang C.T., Lin S.D. (2017). Atomic-scale epitaxial aluminum film on GaAs substrate. AIP Adv..

[68] Silkin V.M., Nagao T., Despoja V., Echeverry J.P., Eremeev S.V., Chulkov E.V., Echenique P.M. (2011). Low-energy plasmons in quantum-well and surface states of metallic thin films. Phys. Rev. B.

[69] Barmparis G.D., Kopidakis G., Remediakis I.N. (2016). Shape-dependent single-electron levels for Au nanoparticles. Materials.

[70] Zubizarreta X., Chulkov E.V., Chernov I.P., Vasenko A.S., Aldazabal I., Silkin V.M. (2017). Quantum-size effects in the loss function of Pb(111) thin films: An ab initio study. Phys. Rev. B.

[71] Ruban A., Hammer B., Stoltze P., Skriver H.L., Nørskov J.K. (1997). Surface electronic structure and reactivity of transition and noble metals. J. Mol. Catal. A.

[72] Kresse G., Furthmüller J. (1996). Efficiency of ab-initio total energy calculations for metals and semiconductors using a plane-wave basis set. Comput. Mater. Sci..

[73] Kresse G., Furthmüller J. (1996). Efficient iterative schemes for ab initio total-energy calculations using a plane-wave basis set. Phys. Rev. B.

[74] Ceperley D.M., Alder B.J. (1980). Ground State of the Electron Gas by a Stochastic Method. Phys. Rev. Lett..

[75] Monkhorst H.J., Pack J.D. (1976). Special points for Brillonin-zone integrations. Phys. Rev. B.

[76] Wolfschmidt H., Baier C., Gsell S., Fischer M., Schreck M., Stimming U. (2010). STM, SECPM, AFM and electrochemistry on single crystalline surfaces. Materials.

[77] Waseda Y., Hirata K., Ohtani M. (1975). High-temperature thermal expansion of platinum, tantalum, molybdenum, and tungsten measured by X-ray diffraction. High Temp. High Press..

[78] Roos P., Bertel E., Rendulic K.D. (1995). Observation of an sp-derived surface resonance on Pt(111) indicating the crucial role of surface-states in photoemission. Chem. Phys. Lett..

[79] Link S., Durr H.A., Bihlmayer G., Blügel S., Eberhardt W., Chulkov E.V., Silkin V.M., Echenique P.M. (2001). Femtosecond electron dynamics of image-potential states on clean and oxygen-covered Pt(111). Phys. Rev. B.

[80] Wiebe J., Meier F., Hashimoto K., Bihlmayer G., Blügel S., Ferriani P., Heinze S., Wiesendanger R. (2005). Unoccupied surface state on Pt(111) revealed by scanning tunneling spectroscopy. Phys. Rev. B.

[81] Hulbert S.L., Johnson P.D., Weinert M. (1986). High-resolution inverse-photoemission study of the Pd(111) surface. Phys. Rev. B.

[82] Schafer A., Shumay I.L., Wiets M., Weinelt M., Fauster T., Chulkov E.V., Silkin V.M., Echenique P.M. (2000). Lifetimes of unoccupied surface states on Pd(111). Phys. Rev. B.

[83] Sklyadneva I.Y., Heid R., Silkin V.M., Melzer A., Bohnen K.P., Echenique P.M., Fauster T., Chulkov E.V. (2009). Unusually weak electron-phonon coupling in the Shockley surface state on Pd(111). Phys. Rev. B.

[84] Herrera-Suárez H.J., Rubio-Ponce A., Olguín D. (2012). Electronic band structure of the Pt(111) surface: An ab initio and tight-binding study-I. Comput. Mater. Sci..

[85] Theileis V., Bross H. (2000). Relativistic modified augmented plane wave method and its application to the electronic structure of gold and platinum. Phys. Rev. B.

[86] Dal Corso A., Conte A.M. (2005). Spin-orbit coupling with ultrasoft pseudopotentials: Application to Au and Pt. Phys. Rev. B.

[87] Glantschnig K., Ambrosch-Draxl C. (2010). Relativistic effects on the linear optical properties of Au, Pt, Pb and W. New J. Phys..

[88] Di W., Smith K.E., Kevan S.D. (1992). Angle-resolved photoemission study of the clean and hydrogen-covered Pt(111) surface. Phys. Rev. B.

[89] Frantzeskakis E., Pons S., Crepaldi A., Brune H., Kern K., Grioni M. (2011). Ag-coverage-dependent symmetry of the electronic states of the Pt(111)-Ag-Bi interface: The ARPES view of a structural transition. Phys. Rev. B.

[90] Henk J., Ernst A., Bruno P. (2003). Spin polarization of the L-gap surface states on Au(111). Phys. Rev. B.

[91] Takeuchi N., Chan C.T., Ho K.M. (1991). Au(111): A theoretical study of the surface reconstruction and the surface electronic structure. Phys. Rev. B.

[92] Kevan S., Gaylord R. (1987). High-resolution photoemission study of the electronic structure of the noble-metal (111) surfaces. Phys. Rev. B.

[93] Crommie M.F., Lutz C.P., Eigler D.M. (1993). Confinement of electrons to quantum corrals on a metal surface. Science.

[94] Hasegawa Y., Avouris P. (1993). Direct observation of standing wave formation at surface steps using scanning tunneling spectroscopy. Phys. Rev. Lett..

[95] Crommie M.F., Lutz C.P., Eigler D.M. (1993). Imaging statnding waves in a 2-dimensional electron gas. Nature.

[96] Li J., Schneider W.-D., Berndt R. (1997). Local density of states from spectroscopic scanning-tunneling-microscope images: Ag(111). Phys. Rev. B.

[97] Petersen L., Laitenberger P., Lægsgaard E., Besenbacher F. (1998). Screening waves from steps and defects on Cu(111) and Au(111) imaged with STM: Contribution from bulk electrons. Phys. Rev. B.

[98] Bürgi L., Jeandupeux O., Hirstein A., Brune H., Kern K. (1998). Confinement of surface state electrons in Fabry-Pirot resonators. Phys. Rev. Lett..

[99] Kliewer J., Berndt R., Chulkov E.V., Silkin V.M., Echenique P.M., Crampin S. (2000). Dimensionality effects in the lifetime of surface states. Science.

[100] Nicolay G., Reinert F., Hüfner S., Blaha P. (2001). Spin-orbit splitting of the L-gap surface state on Au(111) and Ag(111). Phys. Rev. B.

[101] Bansil A., Lin H., Das T. (2016). Colloquium: Topological band theory. Rev. Mod. Phys..

[102] Keimer B., Moore J.E. (2017). The physics of quantum materials. Nat. Phys..

[103] Liu W.E., Hankiewicz E.M., Culcel D. (2017). Weak localization and antilocalization in topological materials with impurity spin-orbit interactions. Materials.

[104] Hasan M.Z., Kane C.L. (2010). Colloquium: Topological insulators. Rev. Mod. Phys..

